# Interrogating the “Us” Versus “Them” Dichotomy in Technology Research with Older Adults

**DOI:** 10.1145/3772318.3791086

**Published:** 2026-04-13

**Authors:** Amanda Lazar, Elissa Carpio, Ruipu Hu, Beth Barnett, Kibron Tesfatsion, Sheena Erete

**Affiliations:** College of Information, University of Maryland, College Park, Maryland, USA; College of Information, University of Maryland, College Park, Maryland, USA; College of Information, University of Maryland, College Park, Maryland, USA; College of Information, University of Maryland, College Park, Maryland, USA; College of Information, University of Maryland, College Park, Maryland, USA; College of Information Studies, University of Maryland College Park, College Park, Maryland, USA

**Keywords:** Aging, Technology trends, Older adults, Us vs Them, Othering, Autoethnography

## Abstract

Research on technology for older people focuses on older people’s experiences–and understandably so. However, the phenomena of othering, or seeing this group as different and worse off, is a persistent problem. In this project, we turned inwards through an 8-month collaborative autoethnography to understand our own experiences with technology issues and supporting others in technology use. We found that each member of our mixed age group faced pervasive and burdensome technology issues and recognized that some of the burden is associated with the evolution of technology tools. Our work contributes an expanded understanding of aging as a sociotechnical process and identifies counternarratives to implicit assumptions we held as HCI researchers working with older people. Our research also shows how reflexive positional methods can surface often unexamined experiences with technology issues and aging among researchers.

## 1 Introduction

Researchers working with older adults in HCI are at a standstill. Over more than a decade, research has eroded the assumption that older adults can be uniformly and distinctly characterized, whether in terms of cognitive and physical abilities, preferences, care needs, or technology use [42]. At the same time, prior work repeatedly shows that older adults are regarded as the “other” by researchers and designers, which can result in the design of unwanted and stereotypical technology [2, 10, 44, 45, 50, 74]. Given that a difference between older and younger people’s technology use had been the ontological premise to much prior research with older adults in HCI [74], researchers are now left asking, “is ‘older adult’ a meaningful category” (page 1-2 [41]).

Past research interrogates the ways that the category of “older adult” is used to make blanket assumptions about the technology use of people over 60 or 65 [2, 10, 54, 60]. However, it does not dive into the lived experiences of the parties “doing” the othering. We are thus missing an understanding of the constructor of the “us” vs “them” dichotomy with older adults–which includes the researcher. In this study, we paused to engage in reflexivity, or an understanding of how our worldview affects the knowledge that we produce [65]. Our study answers the following research questions:

- What can turning inward reveal about the link between aging and technology use?
- What role can reflexive research-centered methods play in disrupting “othering” based on older age and technological competence?

We engaged in an autoethnographic approach over 8 months. Our initial goal was to understand our encounters with technology issues and how we support other people, and older adults in particular, in navigating technology issues. Our findings detail our experience studying technology issues, as well as our introspections on aging in relation to technology. We helped others with a variety of technology issues but also encountered our own issues. These issues ranged in complexity and took place in our homes, at work, and on the go. For some of us, realizing that we faced technology issues was surprising, and led us to contend with uncomfortable feelings about our age and technology competency.

Our contributions are two-fold. Our first contribution is empirical: we found a link between aging and technology issues minimally associated with age-related changes to our bodies (i.e., cognitive, sensory, fine and gross motor changes or chronic conditions). We recognized that over time we are all changing (that is, aging) and, at the same time, technology is constantly changing. Our work thus shows concepts such as “aging as a process” [49] and “situated elderliness” [10] happening much earlier in the life span than our field has considered to date. Our second contribution is methodological. We demonstrate how collaborative autoethnography can serve as a rigorous and reflective method for aging research in HCI. By drawing on a mixed age-group’s lived experiences with technology across varying life stages, we provide a nuanced, insider perspective that challenges dominant assumptions about age and technology use. Our approach foregrounds personal narrative as empirical data that illustrates not only how engaging with one’s positionality can produce rich social, cultural, and technological insights, but also how it can help us reposition and reimagine how we approach aging research.

## 2 Related Work

Research has revealed insights into expected and actual roles of older adults in relation to receiving or giving technical support. In addition, critical research in HCI has long called attention to the predominance of homogenous and deficit-oriented views of the technology use of older adults.

### 2.1 Older adults and technology issues

The recognition of technology issues dates far back as the start of our field. Addressing technology issues has long involved technical support, which early on took the form of telephone-mediated help as a mode of specialized support for supporting issues such as mental health crises [27, 48, 51].

HCI and aging research has attended to how older adults navigate technology issues in their everyday lives [55, 63, 67]. Yet in many accounts, older adults are studied primarily as recipients of help, or those who seek support from more experienced users. This includes studies of family technical support in diverse geographical areas, where older adults are found to rely on younger relatives to learn, troubleshoot, and appropriate digital technologies (e.g., [6, 29, 38, 70, 80]. For example, a study of technology learning in China found that older adults’ adoption of new technologies depended heavily on younger family members, whose roles in the learning process were identified as influencers, supporters, protectors, and monitors [70]. A study in Latin America similarly found that older adults received significant support from younger family member such as grandchildren when adopting digital media tools like mobile phones [32].

Recent work complicates this view by showing that roles are not static. Seo et al.’s study [63] of mobile shopping experiences revealed that when older adults’ self-perceived technology efficacy shifts, their roles can change from learners, to collaborators, to providers of support to others. This study suggests that older adults can adopt more roles rather than solely a passive recipient of technical help when interacting with certain types of technology. Similarly, contrary to the stereotypical view of tech support, another study found that some older adults provide technical support to their peers [36]. At the same time, the identity of the “tech giver” can also shift, as they take on various roles in relation to older adults. For example, people who assist older adults with their technology problems may simultaneously hold the role of a caregiver [55], family members [63, 64], or friends [63]. These roles have the potential to change the nature of tech support. One such role is the caregiver. Piper et al. [55] identified different forms of work caregivers perform when supporting their care recipient’s online activity, including guiding, stimulating, connecting, and protecting. These forms of work emphasize “*empowerment*” and “*independence*” introduced by the unique role they take on as caregivers and go beyond simply resolving technical issues. These overlapping roles shape the very nature of technical support and the ways it is provided.

Historical accounts of domestic support settings show that while people often associate younger age with technical expertise, role changes in support are shaped more by experience and competence than by age alone. As computing became more common in homes during the 1990s, studies found that when users encountered technical issues related to setup, maintenance, and troubleshooting, they often sought technical help from other household members rather than formal services [38, 56, 57]. Researchers identified a “guru”-type role in tech support, who is typically the person in the household perceived to be most knowledgeable about computers or the internet [38]. At first, younger age was highlighted as a characteristic of this guru type as the study found this role was often filled by a teenager. Later research complicates the assumption that younger age equates to expertise, finding that in households with children, the guru was not always the teenager, but rather the person with the most technical expertise [18, 30].

Regardless of the age of the technical support providers, role changes can carry an emotional dimension. Prior work notes the pride or pressure of sustaining an expert identity of a technical giver and the discomfort of admitting technical incompetence [15, 58]. These emotional shifts may influence other behaviors in technical support, such as whether and how people step into, maintain, or withdraw from support roles [15, 58]. In this paper, we acknowledge and analyze the emotions that arise when experiencing technical issues and receiving and giving technology support.

### 2.2 Critical reflections in research on aging

Research with older adults in HCI has been described as having undergone a “critical turn” in the 2010s [42]. Researchers reckoned with the ways that older adults were frequently framed as a homogenous group, uniformly in need of care, and chronically ill [22, 60, 74]. One troubling aspect of these characterizations was that they were a way that designers “other” older adults – or define older people as, “a user group that is clearly separated and different from the rest of the population.” [59]. This kind of categorization is not neutral: “othered” groups like older adults are seen as “worse off” [60], including in their ability to use technology [2, 74].

In response to the phenomenon of “othering” older people, researchers have proposed mitigation strategies such as empowering end users so they can create their own technologies [60]. Researchers have also made strides in developing alternative ways of understanding and supporting older adults’ technology use. Petrie recognized the heterogeneity in the technologies older people were exposed to at pivotal points in their life (in particular, school and working life) [54]. Barros Pena et al. noted how even cultural experiences *before* digital technologies are introduced shape older people’s reception of technologies [8]. The approach we can take from these alternate framings is to recognize *specific technology experiences* different individuals have experienced, including by generation, to better understand older participants and design accordingly [54].

Another perspective argues for a shift from a focus on old age as a *state* to a focus on older adults’ aging as a *process* [49]. Doing so can help researchers support changes older people experience without slipping into a deficit-focused lens. Situated elderliness is another approach that seeks to mitigate deficit-oriented framing, by seeing elderliness emerges in the interactions (or intra-actions [26]) between older people and their environment [10]. People are not elderly all the time or due to some inherent characteristic, but rather the environment and the social milieu contribute to disabling conditions [10, 26, 40]. The direction this past research is taking is well needed. However, our stance is that, given that the researcher is doing, or recognizing, the othering, we also need to turn inwards to make progress on this issue.

## 3 METHODS

In this paper, we conducted collaborative autoethnography to examine our own experiences addressing technology issues as HCI researchers. We describe our systematic reflective process below.

### 3.1 Motivation and Project Formation

Autoethnographies, rooted in interpretivist and post-structural paradigms, enables researchers to reflect their own subjectivity and behavior as an analytical resource [1]. In prior HCI research, autoethnographies have enabled researchers to challenges assumptions and foreground research positionality as a critical part of knowledge projects (e.g., [20, 24, 35, 37, 79]). The critical attributes made this methodology appealing to us, as our research often aims to disrupt the dominant framing of aging.

Brooke^1^ is a non-degree seeking graduate student at our university as part of a program for state residents 60-year-olds and over. She has taken several courses and independent studies and has joined the research lab of Ava, a professor at the university. In November of 2023, Brooke had the idea to inform Ava’s lab, which focuses on technology and older adults, about older peoples’ experiences with technology by writing about the technology issues she and her friend group encountered. She created an entry about her experience using a rideshare technology to experiment with what this could look like. Stacey (another professor and HCI and autoethnography expert) noted the opportunity for a rich autoethnography project. Upon Brooke’s suggestion, we decided to open the project up to anyone who wanted to join from Ava’s research lab. We did so initially to avoid an undesirable dynamic to collaboratively examine a single author’s entries, especially given the backdrop of stereotypes around older adults’ technology use. Stacey, Ava, and two members of Ava’s lab, Emily and Rel, decided to join the project.

### 3.2 Data Collection

We began the project after consulting with the IRB, who determined that the study did not meet the criteria for requiring approval for human subject’s research, given that all data would be collected solely from members of the research team. Next, we collectively began to define our process (e.g., the development of writing prompts, and a timeline for completing them) before writing any additional entries (beyond Brooke’s initial reflection). We created a set of prompts and guidelines for the project (see supplementary material). These prompts and guidelines were meant to be flexible, while also providing authors with some ideas and entry points into writing entries. We agreed that each research team member would determine for themselves whether an item (e.g., a microwave) constituted technology.

Our autoethnographic process involved 1) individually writing reflections about our experiences with technology issues, and then 2) meeting as a group to discuss them, over a period of around 8 months. As we describe in the findings section, it took a significant amount of effort to focus on our *own* experiences instead of others’. To do so, we developed several strategies ad hoc. First, we left comments on each other’s entries to encourage each other to reflect on what the author of the entry was feeling, rather than the person they were helping. An example of such a comment in response to an entry that focused on describing the actions and perceived emotions of the “helpee” was a comment Ava left that said, “how did YOU feel through all of this (Rel, “One Unsuccessful Tech Support Experience”, Sep 2024). Second, when our group discussions started to veer into “othering” (often through conceptualizing our experiences as compared to those of older people’s), a member of the group would often speak up to remind us of the goal of the project and what we could comment on with the data source we had (ourselves). Finally, we suggested to each other entry themes to do in the following month to help turn inward, such that each person had an entry on helping someone else with a technology issue (which many team members tended to do) but also experiencing a technology issue and/or *receiving* support. These efforts continued throughout the study – in other words, we did not “grow out of” the dispassionate observer researcher mode. However, it did become easier to refocus on ourselves and our feelings over time.

**Figure 1:**
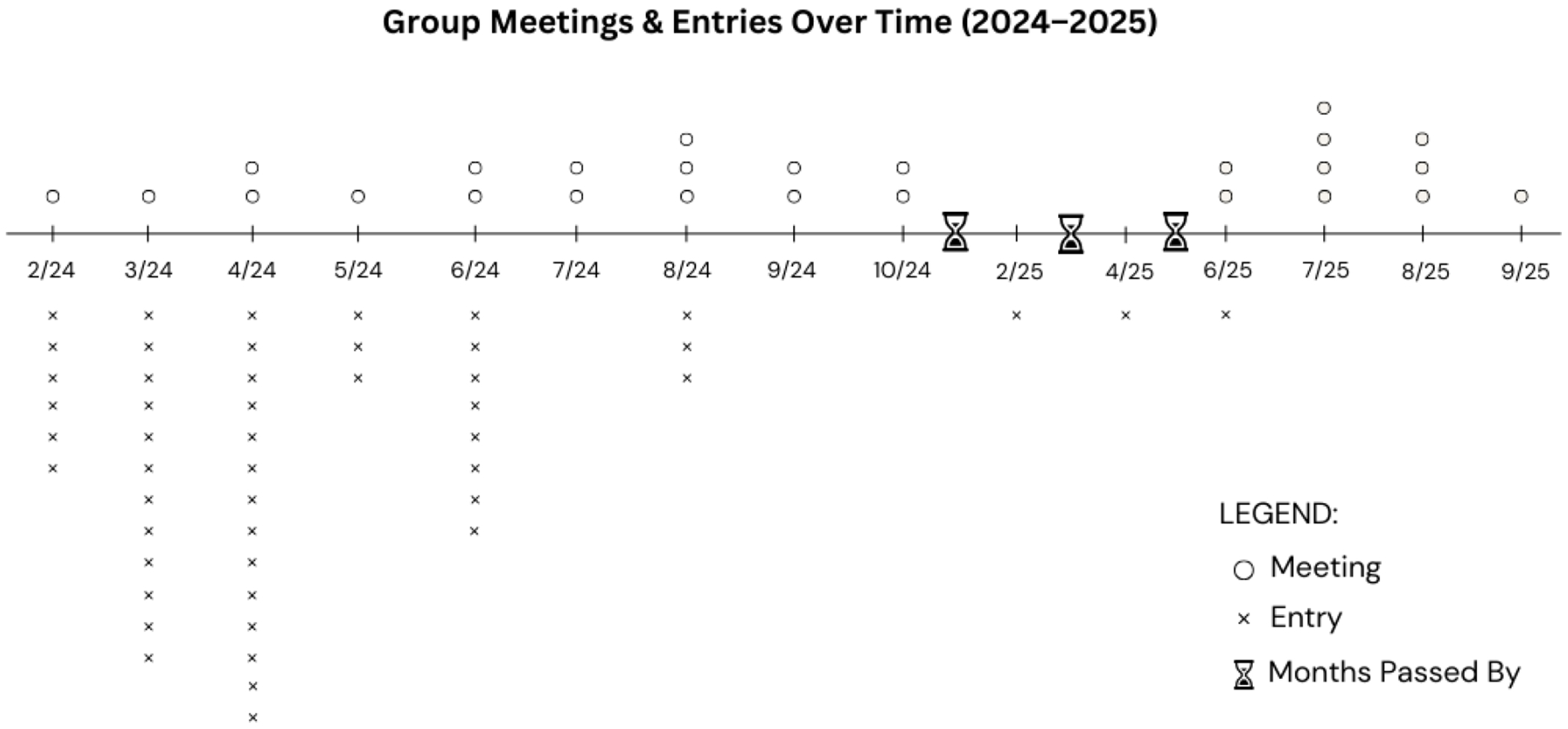
Group Meetings and Entries Over Time (2024-2025). Dates (month/year) and frequency of group meetings and entries. Circles represent meetings and x represents an entry. The clock indicates time has gone past.

Over the 13-month period, we met approximately every other week for eight months, pausing for five months while the lead author was on leave (see Figure 1 for details about the process). There was a total of 26 meetings, and most lasted approximately one hour. Prior to each meeting, we uploaded our entries to a shared drive. During meetings, we read and discussed our own and each other’s entries that had been written during the prior two weeks. While we had developed questions to guide our discussions (see supplementary material), our conversation during the meetings emerged organically as we engaged and reflected on each entry.

### 3.3 Data Analysis

We describe our analysis process as Reflexive Thematic Analysis [11, 12]. We had familiarized ourselves with the entries through reading them before or during each meeting and noting initial ideas as memos or during meetings. Some examples of early memos that became important to our later analysis include “Feeling dated in societal relevance bc of tech issues” and “big emotions!” (Emily, reviewed by team August 2024). When we began our initial coding stage, we uploaded entries and transcripts of meeting recordings on the qualitative analysis platform Dovetail. The first author re-familiarized themselves with the entries and also on meeting recordings, as they read the transcripts of and verified relevant portions of transcripts auto generated by Dovetail. The first author coded most of the transcripts, with the fifth author coding the remainder. While coding, the first author collated codes into potential themes and then began describing these themes in prose. At this point, our group meetings shifted from mostly reviewing entries to mostly reviewing and refining the specifics of each theme, with the first author iterating on themes in between meetings based on each meeting’s feedback and continued coding of the data. When meetings no longer generated new observations or insights, we deemed our analysis (which became findings section 2) complete. For findings section 1, we collaboratively selected attributes of the entries as a whole that we thought would provide readers with enough context to illustrate and provide examples of the themes that emerged. To do so, Brooke created a table with entries and possible attributes of interest to the reader.

### 3.4 Team Composition and Associated Limitations

Of the five team members, two were in their 20s, two between 30 and 40, and one in her 70s. This age diversity became key to many of our insights. We could correct inadvertent generalizations team members made about ourselves or others based on age demographics. Another difference on the team was in the extent to which we felt part of the community studying aging and technology research in HCI. Having individuals with a mix of familiarity on the team helped us question certain assumptions, go deeper to support certain concepts, and explore new avenues.

It is important to note that while we varied in certain characteristics, our team composition could not cover all demographics. Particularly for the CHI community, the fact that we are all US-based researchers means that we are missing important insights, as past research has noted the differing dynamics and needs that exist across worldwide cultural and regional contexts. To illustrate how our regional specificity might have impacted our findings, we provide an example: the desire to be self-sufficient (and some of the negative emotions that followed our inability to do so) likely played into how we wanted to be able to solve our own technology issues, whereas self-sufficiency or independence is a value which researchers have pointed out has been less salient in other cultures, such parts of Latin America [32]. While we hope that some of our findings resonate with others, they are unique to what occurred in our group dynamic at the point in time in which we conducted this project, and we encourage future research to examine other relevant demographics.

## 4 FINDINGS

Over the course of this project, we encountered and documented many kinds of technology issues. Documenting these issues and engaging in continued discussion among our team led us to realize that technology issues are prevalent and burdensome in our lives, and that our membership in the technologically savvy “in-group” was time limited. Our journey to these realizations was not smooth. Accustomed to studying and observing others – older adults – in our research, it took time for us to focus on our own experiences, perceptions, and emotions.

### 4.1 Encountering Technology Issues

Older adults appeared in 28/49 of the entries as help recipients. Though our initial focus was on older adults and technology support, younger people – primarily ourselves – were featured in many of the entries (see Table 1). Across many entries, we found technology issues complex and difficult to untangle (or even communicate in writing). These issues appeared in a surprising range of locations, showing the pervasiveness of technology issues in our lives.

**Figure 3:**
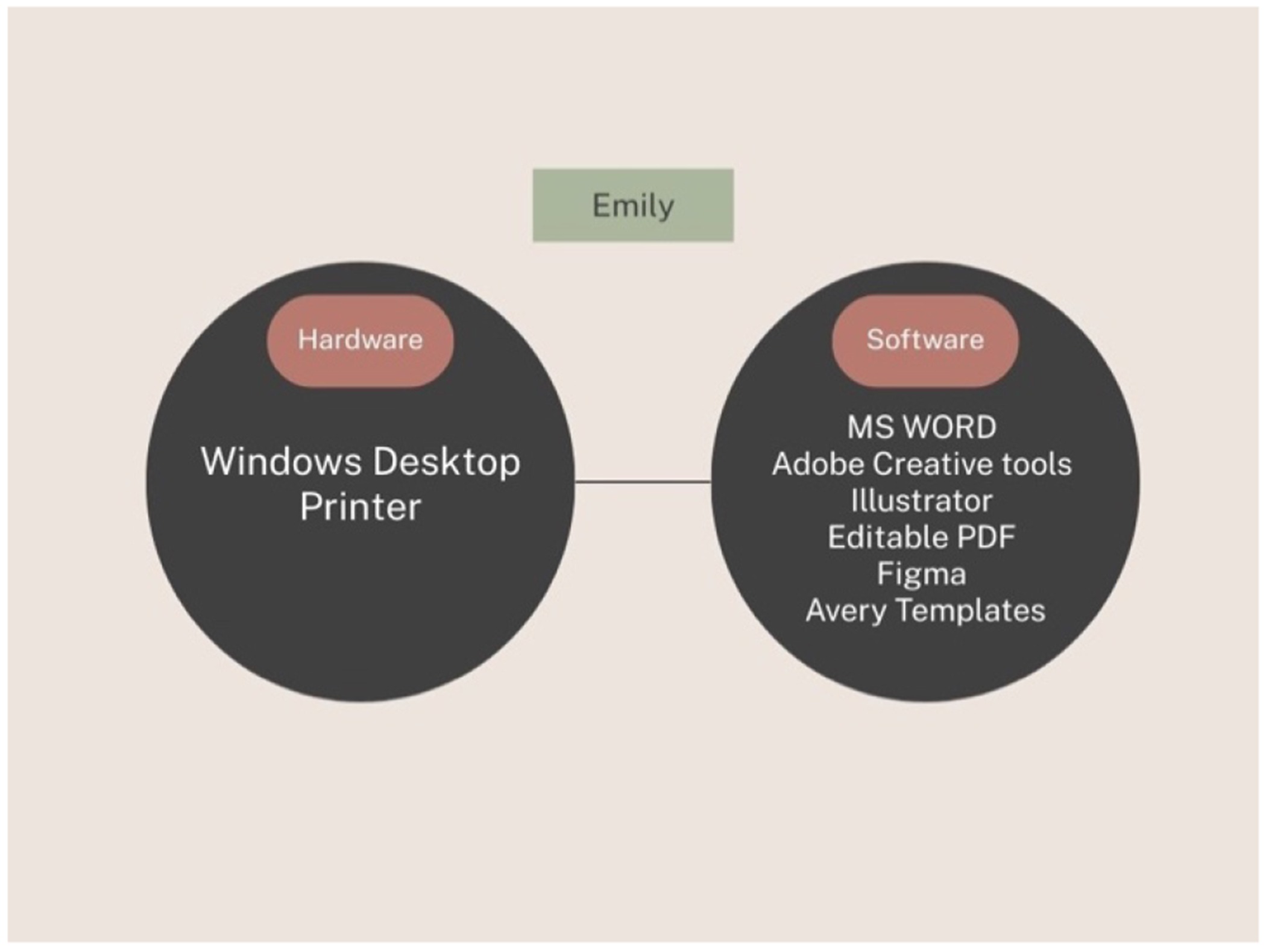
Technologies used by Emily to print labels.

Part of the complexity of technology issues was that approaching them could require a wide range of technologies. When Emily needed to create preformatted name tags with a logo and text, she had to use two kinds of hardware (desktop and printer) but also six different kinds of software to accomplish the task (Figure 3).

Further, technology issues existed across different parts of our lives (see Table 2). We documented encounters with technology issues and support with nuclear and extended family members, peers, and colleagues. Some technology issues were specific to setting, such as home appliances, office work or technology in newer cars. At home, we navigated issues with TVs, microwaves, and smart lightbulbs. Some issues were specific to videoconferencing. A work-related matter included screensharing related to presentations (in the classroom or at a hybrid conference). Most of the issues were not necessarily location specific. It may be that as people work on laptops, phones and other portable devices, and as technology is more and more ubiquitous in daily life, a technology issue can arise anywhere and anytime.

### 4.2 Turning Inwards

Our conception of the relationship between aging and technology issues evolved as we documented our technology issues and engaged in regular discussions among our group. We realized that we are not only helping others with their technology issues – we are facing technology issues ourselves. On reflection, we noticed that our technology issues are a frustrating and sometimes embarrassing burden. This understanding enabled us to comprehend how being in the “in group” with technology is a transient state–something that we all will age out of, if we have not already.

#### 4.2.1 From “them” to “us”: We have (technology) issues.

We are researchers trained in analyzing the ways that other people interact with technology. To engage in the goals of this project, we had to resist the inclination to document and discuss others. As Emily put it, we experienced difficulty in turning ethnography into autoethnography.

Our observations sometimes veered into theorizing about the experiences of other, often older, individuals. This tendency was particularly pronounced when we documented our experiences offering tech support to older people. For example, an early entry by Rel had many inferences about others:

“… I noticed that she (tech support class attendee) always asked questions along with her hypothesized guesses, which were almost right every time. This made me curious about her actions, making me think about breaking down technology use into several steps: first, considering a decision you are about to make, then making the decision, and finally, the decision impacting the interface… the critical part currently slowing [Name] down is the second part…” (“Teaching Videoconferencing to Older Adult Group”, Apr 2024).

We also made generalizations about older adults as a whole, including when Brooke created a distinction between two groups in her entry on “Context & Experience of Tech Life as an Older Adult” (Mar 2024):

“This section of the post (entry) is about how older adults are dependent on technology. My examples are to show that (1) an older adult, like me, can use technology most of the day; and (2) older adults, especially those who have limitations, need technology to keep them in social contact.”

These examples show two divides framed between an in-group and out-group: based on age (under 65 and over) but also between people in the same age category of “older adult” – but with perceived differences in technology ability.

Over time, we improved our ability to critically reflect on when we were veering into imagining the experience of another (people in the entries or people in different age groups more broadly) and shift course accordingly. We continually needed to remind each other of the goal of this project – focusing inwards, on our own experiences (described further in methods). As we did this, we began to have a strange realization. Most of us coming into the project had assumed that the instances of technology issues and support we would find in our daily lives would largely cover our experience helping other people. But through this project, we realized that we were also facing technology issues – and facing them often:

“I always assumed I’ve never run into technology problems, but in fact, I run into them all the time. The other day I was setting up the printer and today I was trying to, you know, just my computer froze for no reason, I’m just trying to restart that. I think that all comes as, you know, problems, but I just never think about that way.” (Rel, Group Meeting, Jun 2024)

“When we first started, like, what kinds of tech issues (do I encounter)? Then I started really thinking and I was like – almost daily.” (Stacey, Group Meeting, Aug 2024)

“… I was definitely I think a little more confident in my tech abilities before we started the project. Because I was blissfully unaware of how much I was running into.” (Emily, Group Meeting, Jul 2024)

Brooke had already reached this realization. After all, she had initiated the project to document technology issues encountered by older people (herself and her friends). The four members of this research group under age 65 had to arrive at the realization that we encountered technology issues and required support just like the older adults that we were so used to working with.

For this autoethnographic project, we had to become comfortable analyzing and sharing our own emotions as part of our internal experiences. We had to resist an ongoing pattern we would fall into of “talking about other people’s feelings” (Ava and Stacey, Group Meeting, Aug 2024). This was an uncomfortable process for some of us. Brooke’s discomfort related directly to the topic: she had purposefully developed her ability to stay in the cognitive domain as a way to solve technology issues:

“It has been very hard to get started writing autoethnography experiences this time (March 2024) because of the requirement that I write about how I feel about experiencing these challenges. I am aware that I have trained, am training myself, to suppress my feelings and to stay in the cognitive side of my brain. Solve the problem. Solve the problem. What can I try next? What resource do I have to solve this problem? So, focusing on feel[ings] runs counter to my work mode.” (“Context & Experience of Tech Life as an Older Adult”, Mar 2024)

On the other hand, Ava linked a discomfort exploring her emotions for the study to broader patterns.

Even those of us who were more comfortable exploring their inner world (Emily described herself as “an emotional person,” and Rel as “feeling proud” to explore his emotions) faced friction in documenting and discussing our emotional state as part of the autoethnographic process. Emily noted about one entry, “I don’t like the Emily that’s coming out in this specific response.” (Emily, Group Meeting, Apr 2024). She said, “I was running into like, I don’t know if I want to share this one (in the meeting), but I was like, I should, I should. It’s part of it. (Emily, Group Meeting, Apr 2024). The last part of Emily’s sentence, “It’s part of it,” is her recognition that we could learn more by being open and vulnerable with the method we employed.

To be open and vulnerable, we had to lose the dispassionate affectation we arm ourselves with in academia. Regarding an entry on her advancing age as it tied to changes in her appearance, Ava shared that she found it “so embarrassing to talk (through an entry) about my appearance, in like a professional setting, especially, it feels very uncomfortable.” As uncomfortable as it was, vulnerability became contagious on our team.

#### 4.2.2 Emotional Recognitions: These Technology Issues are a Burden.

Once we were able to access and share our emotions, we were able to realize how encountering technology issues and asking for support was a frustrating experience. Ava gave context to this when she said, “… a lot of this project has been for me, realizing how hard tech stuff is in my life too … and how mad I get.” [Memo May 21]. Feeling that we could be seen by others in our confusion could compound these negative emotions into anxiety, embarrassment, and shame. And these feelings could become compounded even further when they took place “in a professional setting versus a personal setting” (Emily, Group Meeting, Apr 2024).

We got frustrated or annoyed about a lot of things involving technology – helping older relatives with remotes, microwaves, applying for assistance, and resolving phone issues, but also our own attempts at cancelling subscriptions and medical appointments, accessing patient portals on small phones, getting our headphones and smart light bulbs to work – we have examples for nearly every category of Table 1. When we were facing technology issues, doing so in public appeared to amplify our distress: Emily wrote that she ”immediately mentally panicked” when she didn’t find lever in car to open the fuel tank. She said, ”If the station wasn’t busy, I probably wouldn’t have panicked…I had to sit there in my front seat, very embarrassed, while other cars are waiting, and I Googled ‘Honda Civic 2024 How do I open the gas tank?…I was so grateful that the info was online”.

We described frustration, anxiety, and stress in our professional lives. Our role as practitioners (future for Emily, past for Brooke who provided IT support for an organization with a staff of 50 people), and technology researchers came into play here too. We discussed how our high expectations of ourselves, and perceived expectations of others, could link to how “we work in tech, because like we’re supposed to be good at this (Emily, Group Meeting, Apr 2024). Stacey and Emily described how these feelings of inadequacy led to resistance to getting support when they might have needed it. In a meeting, Stacey explained that she had felt the need to “justify” that she had tried all the right steps to print to a network printer to the IT staff:

“I’m supposed to be a tech person and I can’t print at a printer? I’m the one who fixes the printer in my family. And so the notion that I would not be able to even print, and I’ve read the instructions. I know how to do it. I’ve printed many, many places. I don’t know why I cannot get this thing to print at all. And it’s pretty consistent too. It’s also an embarrassment because like, I think the first two times it’s like, ok, fine. But now it’s like an embarrassment. I feel like I’m the only one^2^ that can’t figure out how to print” (Stacey, Group Meeting, Aug 2024).

As with all of our experiences, not all of us respond the same way to similar-seeming encounters. Ava mused that her issues with the printer did not feel as sensitive, perhaps because she did not have a graduate degree in Computer Science like Stacey does (“there’s other things that I feel more insecure about… which don’t come up very much, like math”). Rel described not having the same sensitivities, sharing an example of formatting a research paper for publication. He said he viewed the technology issues he was facing as “more specialized, that requires special skills to fix those problems, which I’m totally comfortable with me not knowing how to solve those problems and asking for help…Those things. I feel like I don’t need myself to know in a daily basis (Rel, Group Meeting, Jul 2024).

A complementary understanding to the recognition that we face technology issues was that, at times, we need help from others to use technology. Emily explained a shared sentiment: “when I first started the project, I kind of assumed… I’m mostly just gonna be writing about helping other people because that’s what I do.” (Emily, Group Meeting, Jun 2024). In several entries and discussions, we noted the difference in age between us (help receivers) and those who provided support (who Stacey described as often “super young… like college students” (Ava and Stacey, Group Meeting, Aug 2024). We analyze this and other outcomes of relating to people of different ages in depth in the text that follows.

#### 4.2.3 Recognizing the transience of “in group” status.

We had been quite comfortable thinking about ourselves as the sophisticated technology users who helped other, older people. Realizing we have technology issues, and need help using technology, partially diminished this comfort. The recognition of ourselves as aging, and aging in relation to technologies and younger generations, affected us further. We recognized that we could not take our technology savviness for granted. And, we finally (in terms of career-long interests) found some links between aging and our technology issues. Specifically, these links involved technologies continuously changing and our inability to keep up, and new generations who seemingly did not encounter the same issues as we do.

Awareness of our own aging as it links to technology issues came up when we confronted technology interfaces that have changed and are therefore no longer familiar. This tied to an awareness of our age having advanced in a negative way–specifically, a feeling of being “out of date”–for Stacey, Emily, and Ava. Stacey recorded an entry after she had volunteered to help a PhD student set up the projector screen at their dissertation defense, but found, “It does not look familiar to me. It does not look like any of the screens that I have access to… and I have no idea how to turn this on.” She interpreted this situation as this technology having progressed past her understanding of it: “maybe it was more advanced, than I was used to,” and made her “feel, maybe incompetent, maybe dated” (Stacey, “Issues with Projector at Dissertation Defense”, Mar 2024). Emily described a similar feeling with a different technology, once familiar and now appearing alien. Reflecting on her encounter with her new car’s capless gas tank and auto-adjustments during cruise control, she said:

“Feeling old in terms of relevance to what’s ‘in’ is something that I feel much more increasingly with regards to tech. This feeling of: I don’t know how this works, I don’t even know where to look, and I don’t know what is even capable of happening…” (Emily, “Learning Curve for New Car/Tech”, Apr 2024).

We sometimes compared ourselves to younger groups who, we imagined, had formative experiences [54] with newer technologies that we did not. Whom we considered young was often relative to our own age, stated explicitly when Emily described her assumption that “anyone younger than me” would have the technology knowledge that she did not (Group Meeting, Apr 2024). Rel expressed fear about his future rather than his present, imagining not being able to keep up with the evolution of technology compared to other, younger generations. He said,

“I realized technology will always evolve and humans will always evolve…But I would worry about people who are younger will be smarter than me… I see some kids, you know, they’re holding up an iPad. That fear, really, you know, got strong, cause I feel like - Wow. At the age of 6. Really, I don’t even know what this device was when I was 6. And now they’re so fluent with this device language. Tapping, moving. And I just realized people started, or students started to have these AI classes in middle school and kindergarten, those, you know, really make my fear become more realistic…” (Rel, Group Meeting, Aug 2024)

In this quote, we can see Rel’s fear of a future in which he is outpaced and out of date compared to younger, more tech-savvy people. Brooke offered a higher-level perspective as the other members of the team expressed these fears – that this phenomenon is one that continues: “So, Gen Z is supposed to be digitally native and- but the truth is that they won’t be digitally native. But because by the time 20 years from now, what digital means will be different from what it means now” (Brooke, Group Meeting, Aug 2024). This perspective connected to the major issue HCI researchers working with older adults have been trying to address – what does it mean to design for older adults as a category – when she said on August 14th in a meeting:

“… Listening to you all (discussing our mental models being out of date for contemporary technologies), you all are talking about all the things older (people)-we’ve gotten used to. I’m gonna make an argument that it’s fallacious to have technology for older people because you’re talking about what we all have to do, living over years as things change, we have to adapt.”

## 5 DISCUSSION

Engaging in an autoethnography with our mixed age team enabled us to turn from a focus on older adults’ technology issues to our own. Each one of us, regardless of our professional backgrounds, computer science degrees, IT experience, or technology research experience, face issues with technology often. We find ourselves in positions where we need help to solve our technology issues, though we might not have labeled it as such before the study – we certainly would not have thought of our needs as similar to the technology needs that we identify in our research with older adults. Yet our own aging emerged as a relevant factor in our analysis. Though most of our team would not consider ourselves as part of the “aging” or “older” population, we still experienced significant technology issues that we suspect are overlooked because we are not considered to be a part of the “aging” demographic. In this discussion, we reflect on our findings through counternarratives that can advance our field’s conceptualization of aging and offer methodological implications for future aging research.

### 5.1 Counternarratives and Provocations

Erete et al. note that “Autoethnography as a methodology creates counternarratives” [24]. We propose the following counternarratives for researchers working on projects with technology and aging. We situate these counternarratives in the context of some of the internalized narratives of aging that we surfaced over the course of this project. We also provide provocations for researchers who wish to experiment with acting on these counternarratives.

#### 5.1.1 We are all aging.

##### Initial internalized narrative:

In many societies, at a certain age (often 60 or 65), a person is considered to have entered the category of “older adult.” A categorization by age structures “our” (researchers’) ability to study “them” (older people) as a group.

We began the study with an internalized narrative that strictly separated us from the kinds of people we study. This was also true for Brooke, who meets the 65+ qualifications, and sometimes referred to her own technology issues, but often as a comparison to how older adults with less technology literacy might experience these issues. It was much easier, for all of us, to focus on other people’s experiences than our own.

Our engagement in this way can be described as an “othering” process, which separates the dominant group – the “us” – from the others. Past research has largely focused on how to compensate for or mitigate this tendency to “other” older adults. For example, Rogers and Mardsen advise shifting from a rhetoric of compassion for the “other” to one of engagement and empowerment [60]. Ambe advises looking at older people as individuals rather than an amalgamation [2]. While these approaches are vital, they take for granted the legitimacy, or at least the stability, of the dichotomy between the “us” and the “other”.

##### Counternarrative: We are all in the process of aging.

While we may not all meet the age-based criteria of older adult, we are all in the process of aging. When we see ourselves on the continuum of aging, we become aware of the trajectory of change in ourselves, in technology, and in our challenges using technology. The distance between “us” and “them” shrinks and wobbles. We may start designing for our own aging selves as well as others, which could lead to designing less with stereotypes and assumptions and more for persistent, common issues we face related to age. HCI research has long shown the power of communities designing for their own needs and the barriers that they face. We detail some of the commonalities that our researcher communities may have with those we so often study from a distance in the next section.

##### Provocation: Challenge the “us” versus “them”.

“Othering” older adults will ultimately harm us all as we age. While it is tempting to put distance between ourselves and this stigmatized group, it is in our long-term interests to contribute to a society that does not devalue people as they become older.

The dichotomy between “us” (not older adults) and “them” (older adults) is reinforced, and therefore ripe for challenging, in many realms – quick glances at the mirror, interactions with others, and in our scholarship. Internal work can take place when we reframe aspects of aging that seem problematic as strengths, rather than deficits [75]. For example, instead of accepting the idea that we lose value as we age due to changes in our physical appearance, we can focus on how we may get taken more seriously, or that each wrinkle means that we have lived another year. We can also challenge the alienation of older people that happens casually in social conversations by pausing when we or others make jokes about aging and ask, is there something we are self-conscious about here? Can I feel closer to the people I am separating out as older or younger? Finally, we can challenge ourselves to skip the paragraphs at the beginning of a paper or a grant that so often set up the great demographic issues associated with aging and point to the issues that older adults have as if they do not afflict us all (e.g., loneliness, physical health issues).

#### 5.1.2 We all have (technology) issues.

##### Initial internalized narrative: Older adults have technology issues.

Though we have, in our research and mentoring, long critiqued the dominant view of older adults as homogeneously experiencing challenges in the use of technology (along with many in the field e.g., [23, 74]), we were still not exempt from beginning our project with these internalized beliefs. Our internalized age bias as well as our approach to research (understanding where users face difficulties) led us to consider older people as the ones with technology problems^3^. It took us substantial reflection to realize that we even faced something that could resemble the technology issues we so easily find in others’ practices.

As researchers of technology for aging, we and others in our community have not looked in depth at ourselves, centered our own aging identities, or discussed the challenges we experience. In focusing on the other, we have taken for granted our own status as the in-power majority. It is time to question that status.

##### Counternarrative: People with varying technology skills face technology issues across ages. And yet, aging is not independent from technology issues; the aging process does lead to technology issues.

We experienced technology issues often, in many settings and circumstances.

Several researchers have drawn attention to the ways that older adults’ technology use must be understood in terms of the diversity of experiences with, and even before the introduction of, past technology cycles [8, 54]. We learned that aging impacts the technology issues we experience well before we meet the age-based criteria of older adulthood. Technologies, interfaces, and paradigms change over time. We were left feeling “out of date” as we tried to use everyday technologies, like cars and presentation software, that had once been familiar to us but had evolved in form since we had learned to use them. We felt further behind as we observed (or imagined the experiences of) those younger than us, who seem ahead of the curve with these new technologies given the milestones they experience relative to the introduction of these technologies [54], age and become part of our cultural and social networks.

##### Provocation: Frame issues with technology as inherent to technology use, and not exclusive to older people.

As a research community, we have made commendable efforts to address stereotypes of older adults as technologically incapable by showing how older adults are adept at using technology [3, 14, 17, 31, 33, 43, 61, 68, 69, 77] or resist for good reasons [8, 13, 39, 40, 46, 47, 72, 73, 76, 78]. This may inadvertently lead us to gloss over the real usability issues that exist with technologies. One way of addressing these issues without “othering” older adults as a population that is bad with technology is to recognize issues with technology as a far-reaching issue that is not isolated to those of us over a certain age.

If we acknowledge shared issues with technology across generations, we can start making technologies that work better for all of us as we age. We can expand our repertoire past designing special technologies for people over a certain age to more broadly design for those who experience “age-related” technology issues when interfaces, societal expectations, or personal responsibilities change and create mismatches between our technology use and the technologies we have available. We offer a set of provocations to help researchers avoid designing in a space where older adults seem like such a homogenously distant other in terms of their technology use.

#### 5.1.3 We all need (technology) help.

##### Initial internalized narrative: The HCI researcher’s role is to support older people in using technology.

In our cultural and geographical context in the United States, needing help or being dependent can be seen as a negative attribute. Past research has noted defense mechanisms that older adult participants exhibited when they needed technology support during a research study [28]. But some of us also felt embarrassed asking for technology help or had a hard time thinking of ourselves as in need of it.

It seems that we, and most likely many others in the field, have positioned ourselves in the comfortable role of “tech guru” [18, 30]. We realized that we could trace this inclination not only implicitly in our own scholarship, but also to our volunteer technology support work with older adults. Since doing this study, we have reflected about the ways that we feel the need to appear in an expert role even when people bring us problems, for example, with different technology ecosystems or older devices, where we actually do not know what to do.

##### Counternarrative: The HCI researcher will be an expert *offering* support in some situations, and a novice *seeking* support in others.

Our mixed-age group needed support for our technology issues, regardless of our advanced technical degrees or competency in supporting others in using technology.

##### Provocation: Admit support needs.

Presenting ourselves as fellow learners in interactions with participants could shift existing dynamics. In the example of volunteer technology support, instead of presenting ourselves as knowing solutions to any technology problem someone brings us, we could frame the approach as more collaborative – how we are working together to figure out how to fix something. Sometimes the volunteer knows more, but sometimes the person seeking support knows more, for example when they have a different operating system. Working in this more collaborative style will not be without friction – past research has noted that older adults may also expect younger people to be more knowledgeable [5]. However, this same research points out that older participants in their study did not want to be a burden on young users – a collaborative style could help there as well [5].

### 5.2 Methodological Implications

The contributions of autoethnographies are often to bring knowledge to the field that is situated in accounts of shared experiences that go unheard. Autoethnographies in past HCI research have sought to “amplify the voices of those who have been ignored, silenced, or erased” (Erete et al., 2023, pg. 34). This has included firsthand accounts of Black women in HCI [24], a Hard of Hearing traveler, [37] and women using fitness trackers [19].

Experiences like those of our team in this project have not been silenced or erased in our communities. Rather, accounts like ours go unheard because the emphasis in technology and aging research is so often on studying older people, outside of the research team. Through our process, we experienced cognitive and affective shifts from a predominant focus on the experience of the “other” to a focus on ourselves as well as the relationship between ourselves and the “othering” we have done through our research.

We began this paper describing research with older adults as stymied. We hope that our research offers a step out of the conundrum, particularly in pointing out one way that we can emphasize age in relation to technology design without shifting into “othering.” We found that for ourselves, we had not internalized the insights we have gleaned from poring over the literature calling out ageism or stereotyping in HCI [16, 23, 60, 74]. While much ageism and “othering” has been called out, with strategies offered to mitigate it, we had not been able to shift our mindsets. So, despite speaking often about ageism to others, our own internalized biases were alive and well. We needed to engage in long, slow, self-reflection in dialogue with others to start to surface and address them. We still have much work left to do.

Reflecting on the unconscious assumptions and worldviews that shape our research is vital in HCI [62]. Yet our field has been critiqued for limiting this type of reflexive work to a paragraph in the method section describing characteristics of the researchers such as age and race [65], if it appears in our research at all [9]. What if we see the work of identifying our positionality as less about identity markers and more about surfacing deeper reflections on how our perspectives impact how we live, work, and play? It took months for us not only to gather our accounts which show attitudes towards age in action, but also to iteratively reflect in a mixed age group. Seeing reflexivity as a continuous practice helps us engage with it as a living, rather than static, practice–which allows for an understanding of how our worldviews play different roles in forming research questions, recruitment, analysis [44] and every other stage of research, as well as how we can change, shift, or swing back and forth between perspectives [65]. However, we need spaces in which to do this reflexive work.

We recognize that doing this work is not accessible to all. We needed to work in a group where we felt comfortable sharing embarrassing, personal thoughts that contrast with how we would like to think of ourselves or present ourselves to others. We need to have or gain competence in identifying and talking about our emotions [7]. In addition, this is a slow process, and we had the privilege of time to work things out (and we needed that time, as our group went through a mid-study “slump” where we felt worse about our own aging). Other researchers have made headway in identifying slow practices that may be suitable for projects where our approach is not feasible (e.g., [53, 62, 71]). Finally, we cannot generalize our method to working with other “othered” groups, as the harms and societal treatment of them are so different that it may not be wise to have someone in the room with those doing the “othering.” What we found essential to be in the room that might be more accessible was people with an understanding of critical perspectives in the field and people with knowledge of how to conduct critical autoethnographies.

Conditions that can support a reflexive space where these insights can emerge include an iterative approach in community with others. Iteration was necessary because our initial insights started very surface level. As we built trust through meeting regularly, we began to unpack our personal experiences together, which included sharing our insights as they became deeper over time. We were also able to iterate and take time to reflect because our process was not in connection with one particular project, which can feel rushed in academia [25, 34, 66]. While others may not have time to do deep dives through a separate project, integrating this kind of reflexivity into our typical methods in qualitative and design research, such as during memo writing and group discussions of these memos, may be useful. This type of method also opens the door for the creation of various affinity groups of researchers and community members who are not tied to specific research projects allows them to explore these issues without the pressures of a project, funding, and other obligations [23]. While this may require additional work from already burdened researchers and community partners, we believe that participation in such a group would result in richer personal and research experiences. More simply put, our participation in this group has changed not only how we approach our work, but also how we approach our lives.

Working with a community of others was necessary because the self-reflections we shared in individual entries were not enough to get to our insights–many of our insights only emerged when we considered, and pushed and pulled against, others’ shared experiences relative to ours. We need age-diverse teams within to do this kind of reflections, so that we can uncover biases that cut across or are specific to different generations. Working iteratively in community with others is also necessary to for us to keep one another from falling into imagining what other people are feeling. Another attribute that was useful was having a group that was not just academics in the same research area, but also people with industry experience. This helped us look at technology trends differently and go beyond the kinds of points we had made or seen in the past in our writing or workshops with other academics in the space.

## 6 CONCLUSION

People tend to ask after we give talks on technology support for older people, “But will we still need things like this when the current generation ages into 65+?”. This question implies that the group categorized as digitally proficient (the “us” or those a little older than us) will not become the “them,” perhaps because it is so hard to imagine becoming out of date with technology. What we learned in this study is that we have already become the “them,” or that the “them” was never really so different from “us”.

At the beginning of the study, we were often focusing on what the older adults seeking technology support might be feeling. When we encouraged one another to turn inwards, feelings like frustration, annoyance, and embarrassment came to the surface. When we started to consider tech issues we routinely encounter, we found that we too experienced the emotional burden.

We experienced the concept that aging is a process, not a status; at any age we can be confronted with changed technology and new, unfamiliar technology.

Technology too is changing, not static. It keeps moving past the date we learned it. Technologies we took for granted like cars have dramatically changed since we learned to use them, now featuring tablet-like screens and a touch screen interface, integrating phone services into the car, and adaptive cruise control. In addition, interfaces are constantly changing. It seems that people are experiencing feeling out of data at much younger ages with the current pace of AI development.

We hope for our work to open opportunities associated with recognizing the burden that changing technologies places on users of all ages.

## Supplementary Material

Protocol

## Figures and Tables

**Figure 2:**
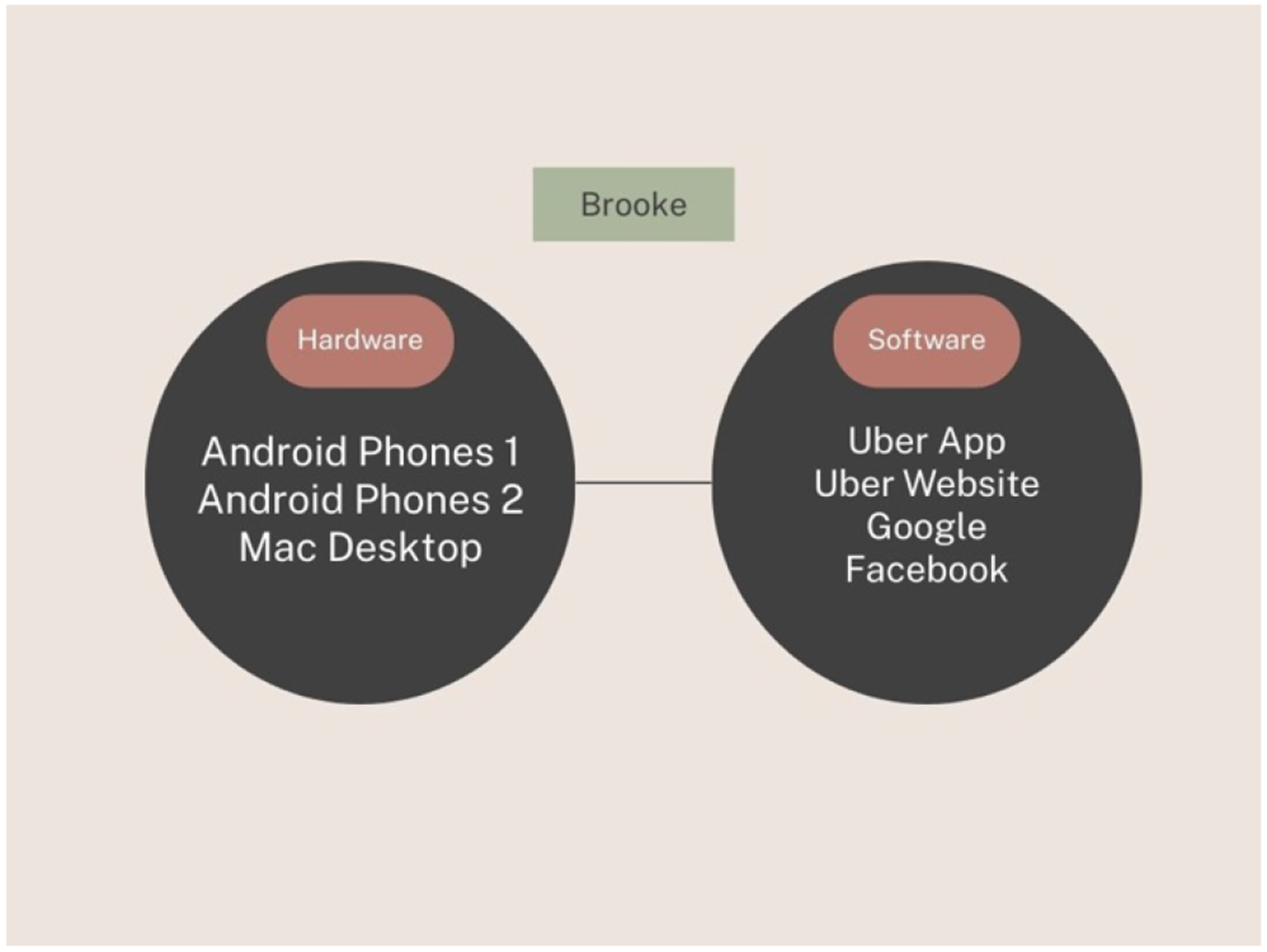
Technologies used by Brooke to change the phone number for her rideshare account

**Table 1:** Technology support interactions by person

Person	Entries	Relationship Type	Receivers age 65+	Involves Professional Support
Rel	5	Peer/Colleague (4), Self (1)	5	1 out of 5
Ava	14	Family (2), Peer/Colleague (1), Self (9)	0	2 out of 14
Brooke	14	Friend (5), Peer/Colleague (1), Self (6)	14	1 out of 14
Emily	12	Family (7), Peer/Colleague (3), Self (5)	7	1 out of 12
Stacey	4	Family (2), Self (2)	2	1 out of 4

**Table 2:** Type of technology in entries

Category (count)	Topics
Residential technology (3)	**Tech:** Microwave, smart light bulb, TV/remote *example:* TV viewers with 3 remotes struggle to find a show using the browse menus; solved by demonstrating the search tool.
Learning a new technology (6)	**Tech:** New audio transcriptions tools, Zoom, eSIM setup for travel, Android phone migration, Amazon KDP publishing platform, new car interface (capless gas tank, cruise control) *example:* New car came with unfamiliar features such as the capless gas tank closure and had to ask GenAI how to open it; the cruise control also worked differently than expected.
Connecting (in)compatible devices (4)	**Tech:** Wireless keyboards, wireless printers, headsets/ear pods, SD cards and card readers *example:* Connecting a wireless keyboard turned out to be surprisingly complicated. The instructions were unclear, and it was uncertain whether the issue was interference with other hardware or an existing Bluetooth connection.
Communication with a business (8)	**Tech:** Canceling online subscriptions, warranty replacement forms, scheduling government appointments, updating account information with companies, applying for financial assistance, professional certification forms, credit card billing inquiries *example:* Canceling an online subscription was difficult. The online option did not work, and sending an email wasn’t fast enough to stop the autopay. A refund couldn’t be requested, and cancellation was only possible after the company later added an online canceling feature to the app.
Communication with healthcare (3)	**Tech:** Canceling doctor’s appointments, accessing lab results via patient portal, scheduling urgent specialist visits across multiple offices *example:* An urgent scheduling problem with a cardiologist could not be resolved through the patient portal, since the doctor worked at multiple offices and messages were not delivered when the doctor was at a different location. And the relevant office had also recently moved, and its phone system was not yet set up, making contact even more difficult.
Office work related (3)	**Tech:** Updating Canva files, creating preformatted name tags, printing to office network printers *example:* Updating a Canva file with this year’s data was unexpectedly difficult. Had to switch to Excel, adjusting colors to match, and then re-importing the result back into Canva to fix a single slide.
Authentication including identity management (4)	**Tech:** Multi-factor authentication for online payments, managing recurring meeting access, handling multiple online accounts, car dashboard requiring dealer-set passwords *example:* A Google One subscriber with multiple accounts was unable to authenticate when trying to update the credit card expiration date, as the system repeatedly failed to verify the correct account.
Presentation (3)	**Tech:** Setting up laptops for classroom screen sharing, managing unfamiliar podium control panels, configuring multi-screen conference presentations *example:* In a classroom, the instructor faced an unfamiliar control panel at the podium while trying to set up a laptop for screen sharing. With no instructions available and little time before class started, there was no opportunity to call tech support.
File Management (3)	**Tech:** Locating files on Google Drive, searching across platforms, handling multiple audio file formats *example:* On Google Drive, a user could not see the full file name and struggled to identify the correct document. Solved by zooming out.
Videoconferencing software (3)	**Tech:** Basic software questions via remote tech support, logging and setting up with in-person assistance for *example:* a tech support expert provided step by step instructions through logging into a Zoom meeting
Security software (2)	**Tech:** Ambiguous security software prompts, uncertainty about when to seek help, limits of peer support *example:* Received an action prompt from security software asking whether to stop notifications, but the message was so ambiguous that there was no clear way to understand or clarify the request.
Other (7)	**Tech-related:** Fatigue from remote work, reliance on phones versus alert devices, dependence on technology in aging, reluctance to give tech help, questions about intuitive design, visibility concerns in online meetings *example:* In Zoom meeting, if want a bigger font to read a shared screen, is that related to aging or seen by others as indication of aging?

**Table 3:** Counternarrative: We are all aging

Initial internalized narrative	Counternarrative	Provocation
We study older people	We are all aging	Challenge the “us” versus “them” • Reframe negative messaging about aging that you apply to yourself • Notice what jokes about aging “do” – do they help you navigate uncomfortable emotions, manage others’ impressions? • Avoid starting papers and grant proposals by invoking the great challenges associating with population aging, and use language that is easier to see commonalities between people of different ages

**Table 4:** Counternarrative: We all have technology issues

Initial internalized narrative	Counternarrative	Provocation
Older adults have technology issues	People experience technology issues across age – though the passage of time does lead to additional issues given fast technology cycles and changing needs.	Frame issues with technology as inherent to technology use • Interview yourself and your research team with your protocols. Find commonalities and see if this helps you reframe how you think about tech issues. • Write a paper or poster based on data with older participants in a way that does not terms referring to older adults in the title or findings. Notice how this changes the way you interpret the findings. • When writing about older adults’ issues with technology in the framing of a paper, start with shared language to minimize framing older people as so different than others. E.g., “Many people experience with loneliness, and technologies are seen as playing a potential role in supporting isolated individuals. Older adults are no exception…”.

**Table 5:** Counternarrative: We all need technology help

Initial internalized narrative	Counternarrative	Provocation
Our role is to support older people in using technology	We will fluidly move between the roles of giving and needing technology support	• Admit support needs • Modulate confidence when in a tech support or tech expert role and see how the interaction unfolds.

## CCS Concepts

• Human-centered computing; • Human-Computer Interaction (HCI).; • Age;; • Seniors;;

## Footnotes

1 All names in this paper are pseudonyms

2 She is not the only one – “No one can! I have an entry about that too!” (Ava, Aug 21 Ava & Stacey)

3 In fairness to ourselves and others, there certainly appear to be differences in technology use by age (e.g., [4, 21, 52]). But no research that we are aware of has found that people below age 65 do not experience any technology issues. However, this assumption seems to have become implicit – for example, it was very hard to use parallel terms with the people we study, like “technology issues,” for ourselves.
