# Supplementary material for: Interrogating the “Us” Versus “Them” Dichotomy in Technology Research with Older Adults
Source: Proc SIGCHI Conf Hum Factor Comput Syst. Author manuscript; Available in PMC 2026 Jun 2. (PMC13225188; doi:10.1145/3772318.3791086)
Supplement: Protocol [file NIHMS2167563-supplement-Protocol.pdf]

Supplementary Material: Prompts and guidelines

Prompts

- How did I feel? How/did my feelings affect what I did?
- (When offering technology support) How do I think the other person felt? How did that affect my actions and how I supported them?
- What am I assuming about what the other person knows? How does this affect my actions?
- How did I decide what next step to take?
- What are my goals with what I am doing?
- How did what I have learned or experienced in the past affect what I am doing or experiencing now?

Include the following description in each entry:

- What happened
- What we are trying to figure out
- Pictures and details when possible
- What devices are you using
- Where are you getting support (customer service on the phone?)
  Musings/memos (in italics…)

What will trigger an entry?

- When we are trying to figure out a tech barriers and issues?
- When we are trying to help someone else figure out a tech barrier and issue?
- (Researcher note: Observing our own processes of receiving support was added later in the process, see Methods section)

Questions to ask of our groups' entries:

1. Are the issues we are seeing different between tech givers vs tech receivers? Are they different kinds?
2. Can we parse out differences like issues vs barriers vs not knowing about something
3. Are our goals different than large tech training initiatives?(Relational, personal)
4. What are stereotypes about aging that are entries speak to?
5. How does documenting our own tech issues affect the way we look at other people's tech issues?
